# A systematic review of data and models for predicting food flavor and texture

**DOI:** 10.1016/j.crfs.2025.101127

**Published:** 2025-06-26

**Authors:** Michael Gunning, Ilias Tagkopoulos

**Affiliations:** a Department of Computer Science, University of California, Davis, 95616, USA; b Genome Center, University of California, Davis, 95616, USA; c USDA/NSF AI Institute for Next Generation Food Systems (AIFS), USA

**Keywords:** Taste, Flavor, Odor, Texture, Machine learning, Artificial intelligence, Review

## Abstract

This review systematically examines the current landscape of data resources and computational models for predicting food flavor and texture. Taste is the most well-defined sensory component, and molecular classification is aligned with the five basic tastes: sweet, sour, bitter, salty, and umami. Odor prediction, while similar in premise, faces greater challenges due to the vast and diverse range of detectable odors and a lack of standardized olfactory metrics. Machine learning models, including graph neural networks and deep learning methods, have shown promise in identifying taste and odor compounds. Texture prediction has seen comparatively less research interest but may prove to be impactful in food quality control pipelines, although more work is needed in creating robust food texture datasets. The review highlights the growing availability of specialized databases which support the development and benchmarking of predictive models. Despite recent advancements, gaps remain in mapping sensory spaces and incorporating receptor-level data. Future directions include creating more extensive and high-quality datasets, improving model explainability, and exploring innovative applications in food design, fragrance, pharmaceuticals, and environmental monitoring. This work provides a comprehensive resource for researchers aiming to advance the field of flavor and texture prediction.

## Highlights

• 28 total data resources for taste, odor, and texture data are compiled and examined.
• 42 sensory prediction models are compiled, along with performance statistics and details.
• Rigorous definitions are identified for taste, odor, flavor, and texture to disambiguate future research.
• Recommendations are given for future food sensory research and knowledge gaps are identified.

## Graphical abstract

**Figure:**
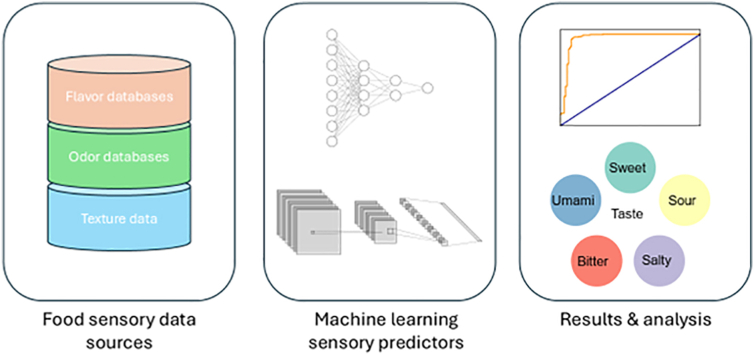

## 1 Introduction

The sensory experience of food is multifaceted, encompassing flavor, texture, and visual appeal (Gravina et al., 2013). Taste, odor, and flavor are well-established drivers of consumer food preferences (Clark, 1998), (Boesveldt et al., 2018), (Boesveldt and de Graaf, 2017), (Liem and Russell, 2019). Understanding how these elements influence palatability is therefore of great interest to the food industry and crucial for research into nutrition and healthy eating behaviors. (Tepper and Barbarossa, 2020), (Beauchamp and Mennella, 2009).

“Taste” and “flavor” are often used interchangeably, even in some scientific literature. However, taste is purely the sensory component that is experienced with the tongue, whereas flavor perception is a complex, multisensory process that has been described as “the sum of perceptions resulting from stimulation of the senses by the food at the junction of digestive and respiratory tracts (Sánchez-Rodríguez et al. Yahia, 2019).” This includes a food's taste, aroma, as well as chemesthetic sensations such as spiciness or astringency. It is the combination of all these sensations that lead to what we experience as flavor (American Chemical Society, 2013), (ISO 5492). Rigorous definitions of taste, flavor, odor, and texture have been sourced from the International Organization for Standards (ISO) and are presented in Table 1.

**Table 1:** Definitions.

Name	Definition	Source
*Taste*	sensations perceived by the taste organ when stimulated by certain soluble substances	(ISO 5492)
	*Examples: sweet, bitter, sour, salty, umami*	
*Odor*	sensation perceived by means of the olfactory organ in sniffing certain volatile substances	(ISO 5492)
	*Examples: fruity, earthy, minty*	
*Flavor*	complex combination of the olfactory, gustatory and trigeminal sensations perceived during tasting	(ISO 5492)
	*Examples: sweet, fruity, minty*	
*Texture*	all of the mechanical, geometrical, surface and body attributes of a product perceptible by means of kinaesthesis and somesthesis receptors and (where appropriate) visual and auditory receptors from the first bite to final swallowing	(ISO 5492)
	*Examples: Hardness, chewiness*	

Definitions of key sensory properties including taste, odor, flavor, and texture. Examples are provided as well.

Within this multifaceted experience, taste plays a fundamental role and is perhaps the most well-defined component. Taste is also straightforward to classify. Scientists have identified five types of taste receptors corresponding to what are known as the “five basic tastes”: sweet, sour, bitter, salty, and umami (Gravina et al., 2013). The taste of a molecule can therefore be expressed as one or more of these categories, based on which taste receptors that molecule interacts with.

On the other hand, odor is not as easily broken down into categories, and there is no agreed upon quantitative measure of odor. Our sense of smell can be described as a measurement of molecular properties, but the specific properties being measured are ill-defined (Wise et al., 2000). As such, in measuring odors, we often must rely on a variety of subjective descriptions such as “earth, fruity, sweet,” among others. Therefore odor, and therefore flavor in general, tends to be more difficult to express than taste alone (Sell, 2006).

Finally, food texture is often cited as a key consideration for consumers and is closely linked to their overall perception of food quality (Bourne, 2002), (Szczesniak and Kahn, 1971a), (Foegeding et al., 2011), (García-Segovia et al., 2008). Texture has been defined as “the sensory and functional manifestation of the structural, mechanical and surface properties of foods detected through the senses of vision, hearing, touch and kinesthetics (Szczesniak, 2002a).” Texture is typically measured by Texture Profile Analysis (TPA), a quantitative measure of various textural properties originally defined in 1963 (Szczesniak, 1963). Since then, there have been some updates to the properties measured by TPA and additional properties have been added to measure liquids. There are five primary properties measured by TPA as well as three secondary properties. The five primary properties include hardness, cohesiveness, viscosity, springiness, and adhesiveness and the secondary properties are brittleness, chewiness, and gumminess. These properties are all well-defined and include reference foods for different measurements, for example hardness is defined as the “force required to compress a substance between molar teeth (in the case of solids) or between tongue and palate (in the case of semi-solids) (Szczesniak, 2002a).” Reference foods for hardness include cream cheese, which is at the lowest end of the scale with a 1, all the way up to rock candy, which has a hardness of 9 (Szczesniak et al., 1963).

In terms of prediction, the current research into taste and odor prediction tends to focus on molecules within specific foods, where the prediction goals may be more narrowly defined. For example, a study predicting flavor compounds in beer classified molecules as aromatic, bitter, sulfury, or other. These categories were derived from standards used by the International Beer Flavor Terminology, and allowed for a more narrow and specialized scope of flavor prediction (Wang et al., 2021), (Meilgaard et al., 1982). Random forests optimized by genetic algorithm were used to develop a multi-objective taste classifier, capable of classifying molecules as sweet, bitter, umami, and other (Androutsos et al., 2024). Deep learning methods have shown promise in both taste and odor classification. In particular, graph neural networks (GNN) have shown superior performance in identifying bitter chemicals (He et al., 2024) as well as achieved human-like performance in identifying odorants (Lee et al., 2023).

Texture prediction research tends to be scarce and the research that does exist focuses on individual foods often with small datasets. One of the challenges in texture prediction is ensuring that the predictive method offers distinct advantages over traditional TPA. While TPA is a well-established and cost-effective approach, it requires physical interaction with the material, limiting its utility in situations where non-destructive testing, rapid analysis, or large-scale assessments are necessary. For a texture prediction method to be practical, it should achieve similar or better accuracy to TPA and provide added benefits, such as faster processing or remote analysis capabilities. Existing research highlights the potential of methods like hyperspectral imaging (HSI), combined with machine learning algorithms, as rapid and non-destructive tools for texture analysis. These methods could enhance quality control processes and boost consumer satisfaction (He et al., 2014), (Zhou et al., 2020), (Cao et al., 2023).

Finally, we present a landscape of current and potential future applications for taste, odor, and texture prediction, highlighting their importance and relevancy to various key areas of food research. In recent years, artificial intelligence (AI) has found many food science applications including diet recommendation (Eetemadi et al., 2020), building food ontologies (Youn et al., 2020, Youn et al., 2024), and nutrient prediction (Naravane and Tagkopoulos, 2023). Predictive modeling of the sensory space provides a more cost-effective approach for food research and development; a field the US Department of Agriculture (USDA) estimates the food industry spends billions of dollars per year on (Agricultural and Food Research and). Other applications include but are not limited to environmental monitoring, food quality monitoring, and personalized nutrition.

While this is not the first review paper to examine machine learning sensory models, we aim to be the most comprehensive resource in terms of data and models. This is also the only review identified that provides rigorous definitions of taste, odor, flavor, and texture, terms which are often conflated or misused. This is also the only review identified that catalogued food texture prediction. In “A survey on computational taste predictors,” Malavolta et al. compile a respectable list of taste, odor, and flavor data, as well as taste predictors. However, our review is more comprehensive in both data and models. No odor predictors are considered, and the word “taste” is sometimes misused in place of flavor. There is also less focus on potential real-world applications (Malavolta et al., 2022).

In “Recent advances and application of machine learning in food flavor prediction and regulation”, authors Ji et al. provide a list of 32 taste, flavor, and odor predictors as well as a discussion comparing different prediction methodology as well as applications. Nonetheless, some shortcomings are that no distinction is made between taste, odor, and flavor, no datasets are included, and texture is not discussed (Ji et al., 2023).

“A Comprehensive Comparative Analysis of Deep Learning Based Feature Representations for Molecular Taste Prediction” by Song et al. is similar in scope to our review but instead focuses on comparing and analyzing different approaches for taste prediction. While it provides a robust discussion of the pros and cons of different taste prediction methods, the scope is limited to methods rather than a comparative analysis of different studies. The focus is also solely on taste prediction and includes no discussion of odor or texture (Song et al., 2023).

The purpose of this paper is to provide a thorough review of all taste, odor, and texture predictive models, as well as to compile the available data resources for training such models. By consolidating information on available datasets, we aim to provide researchers with a valuable resource for future model development and benchmarking. This will include an assessment of data quality, coverage of different food types, and the completeness of sensory descriptors. In doing so, we hope to elucidate research gaps and identify future areas of interest for machine learning in food flavor and texture research.

## 2 Data

There are a growing number of resources containing molecular taste and odor information. We examine their scope, content, and functionality, as well as highlight the overlap between different databases. Table 2 contains a list of all catalogued databases, the number of compounds in each, as well as where to find the resource. Large datasets are critical for building predictive models. Specifically, a substantial and diverse range of data is essential for training models that can accurately predict the taste or odor of novel compounds. Model generalization is also a key factor, as it ensures the model's utility beyond the specific molecules it was trained on, making it a valuable tool for broader chemical discovery and understanding (see Table 3b).

**Table 2:** Data sources.

Name	Domain	Labels	Data quantity	Available at	Pyrfume
FooDB (FooDB)	Odor/taste	Various	70,926	www.foodb.ca	N
Flavornet (Arn and Acree, 1998)	Odor/taste	Various	738	https://www.flavornet.org/	Y
Fenaroli's Handbook of Flavor Ingredients (Burdock, 2009)	Odor/taste	Various	1107	Book	N
FlavorDB (Garg et al., 2018)	Odor/taste	Various	25,595	https://cosylab.iiitd.edu.in/flavordb2/	Y
PungentDB (Chen et al., 2024a)	Odor/taste	Pungent, various	205	http://www.pungentdb.org.cn/home	N
SuperScent (Dunkel et al., 2009)	Odor	Various	2147	Unavailable	N
OlfactionBase (Sharma et al., 2021a)	Odor	106 primary odors, 572 sub-odor types	5109	https://olfab.iiita.ac.in/olfactionbase/	Y
GoodScents (The Good Scents Company - Flavor et al.)	Odor	Various	4565	http://www.thegoodscentscompany.com/	Y
Leffingwell (Database of Perfumery Materials & Performance)	Odor	Various	3522	http://www.leffingwell.com/flavbase.htm	Y
AromaDB (Kumar et al., 2018)	Odor	357 odors	1321	https://bioinfo.cimap.res.in/aromadb/	Y
*Perfume and flavor chemicals: (aroma chemicals), Vol 2* (Arctander, 1969)	Odor	Various	3102	Book	Y
*Atlas of odor character profiles* (Dravnieks, 1992)	Odor	Various	138	Book	Y
IFRA Fragrance Ingredient Glossary (The International Fragrance Association)	Odor	Various	1060	https://ifrafragrance.org/priorities/ingredients/glossary	Y
Sigma-Aldrich (SAFC, 2014)	Odor	Various	1145	https://www.sigmaaldrich.com/US/en/products/chemistry-and-biochemicals/flavors-and-fragrances	Y
BitterDB (Wiener et al., 2012)	Taste	Bitter	1041	https://academic.oup.com/nar/article/47/D1/D1179/5144134?login=true	N
ChemTasteDB (Rojas et al., 2022)	Taste	Sweet, bitter, umami sour, salty, tasteless, non-sweet, multitaste and miscellaneous	2944	https://zenodo.org/records/5747393	N
SuperSweet (Ahmed et al., 2011)	Taste	Sweet	21,980	Unavailable	N
BIOPEP database of sensory peptides and amino acids (Minkiewicz et al., 2019)	Taste	Bitter, sour, sweet, salty, umami, bitter-suppressing, sour-suppressing, astringent	582	https://biochemia.uwm.edu.pl/biopep-uwm/	N
UMP442 (Charoenkwan et al., 2020b)	Taste	Umami, non-umami	442	https://github.com/Shoombuatong/Dataset-Code/tree/master/iUmami	N
BTP640 (Charoenkwan et al., 2020a)	Taste	Bitter, not bitter	640	https://github.com/Shoombuatong/Dataset-Code/tree/master/iBitter	N
SweetpredDB (Goel et al., 2023)	Taste	Relative sweetness	671	https://github.com/cosylabiiit/SweetpredDB	N
PlantMolecularTasteDB: A Database of Taste Active Phytochemicals (Gradinaru et al., 2022)	Taste	Bitter, sweet, sour, salty, umami, pungent, astringent	1527	https://www.ncbi.nlm.nih.gov/pmc/articles/PMC8789873/	N
UMP789 (Zhang et al., 2023)	Taste	Umami, non-umami	789	https://github.com/katouMegumiH/Umami.github.io/tree/main	N
UMP499 (Qi et al., 2023)	Taste	Umami, non-umami	499	https://github.com/daibiaoxuwu/umamiMRNN_datasets	N
SweetnersDB (Chéron et al., 2017b)	Taste	Relative sweetness	316	https://www.sciencedirect.com/science/article/pii/S0308814616317964#s0055	N
Carp TPA (Cao et al., 2023)	Texture	Springiness, cohesiveness, resilience, hardness, brittleness, adhesiveness, chewiness	387	https://www.mdpi.com/2304-8158/12/17/3154	N
Plant-based meats TPA (Kircali Ata et al., 2023)	Texture	Hardness, chewiness	54	https://www.mdpi.com/2304-8158/12/2/344#app1-foods-12-00344	N
Non-fat yogurt TPA (Batista et al., 2021)	Texture	Firmness, cohesiveness, adhesiveness, gumminess	13	https://www.sciencedirect.com/science/article/pii/S0960308521000043	N

A list of taste, odor, flavor, and texture data sources, including their size, availability, and whether they are catalogued in the Pyrfume Project.

### 2.1 Taste

Databases in this section exclusively catalogue tastes. Databases contain either chemicals or peptides and an associated taste. “Taste” is defined as previously, referring to one or more of the five basic tastes: sweet, sour, bitter, salty, and umami. Although sweet and bitter are well represented within these databases, umami data is less common. Sour and salty labeled chemicals are rarer still, with no databases found that exclusively catalogue chemicals with these tastes. Additionally, most taste data available in databases is strictly categorical. Predicting the intensity of a given taste requires quantifiable data, which is currently scarce. Only two datasets with quantitative data were identified, both of which contain relative sweetness for a few hundred chemicals.

**BitterDB.** BitterDB is a database of 1041 bitter-tasting molecules. They also include an associated bitter taste receptor for 270 bitter molecules. Originally made available in 2012, it was updated in 2019 with additional compounds as well as quantitative data such as effective concentration thresholds. Data was sourced from the Merck Index, Fenaroli's Handbook of Flavor Ingredients, as well from various sources in literature. BitterDB also allows users to upload new molecules with bitter taste profiles that are then manually reviewed by the authors. However, at the time of writing, there are still only 1041 compounds listed in the database. Molecules are indexed by CAS number and molecular structure information sourced from PubChem is included as well (Wiener et al., 2012).

**SuperSweetDB.** SuperSweetDB is a large database of sweet tasting molecules. It is made up of over eight thousand compounds, including carbohydrates, proteins, d-amino acids, and artificial sweeteners, and provides a quantitative sweetness value for each. Structural information is also provided, and users can search by similarity based on structure. All data is derived from literature review as well as adopted from existing databases, including PubChem, the PDB and MonoSaccharideDB (Ahmed et al., 2011).

**BIOPEP.** The BIOPEP database of sensory peptides and amino acids is a subset of the BIOPEP-UWM dataset, a dataset of bioactive peptides and amino acids. 582 peptides are sourced from literature as well as various curated databases, including BitterDB, FooDB, and various others. Each peptide is classified as one of bitter, sour, sweet, salty, umami, bitterness suppressing, sourness suppressing and astringent. Each entry in the database includes a reference to the original source claiming its sensory profile (Minkiewicz et al., 2019). This database has been used as a starting point for many peptide taste prediction models, and many of the peptide datasets listed source primarily from BIOPEP, albeit augmented with additional peptides found in literature. Examples include UMP442, UMP499, and BTP640 (Charoenkwan et al., 2020a), (Qi et al., 2023), (Charoenkwan et al., 2020b).

**ChemTasteDB.** ChemTasteDB contains taste information for 2944 unique compounds. Each compound is classified into one of nine taste categories, including sweetness, bitterness, umaminess, sourness, saltiness, non-sweetness, tastelessness, multitaste, and miscellaneous. All data were sourced from various literature and the database is fully referenced. The authors state that the database may be updated with additional tastants in the future, although at the time of writing, there are still 2944 compounds listed in the latest version of the database (Rojas et al., 2022).

**Plant Molecular Taste Database.** The Plant molecular Taste Database is a database of phytochemicals, or specifically phytotastants. Multiple taste databases, such as BitterDB and SuperSweetDB, were scraped for phytochemicals. Additionally, phytochemicals with known taste responses were sourced from literature. The database contains 1527 phytochemicals categorized as bitter, sweet, sour, salty, umami, pungent, or astringent (Gradinaru et al., 2022).

**Quantified Taste Data.** Quantified taste data is data where instead of just a categorical label, a value is used to quantify the relative intensity of a taste. For example, with quantified sweetness data, we know not only if a chemical is sweet, but also how sweet it is compared to other sweet chemicals. This kind of data remains relatively rare compared to categorical taste data but can have important practical applications such as identifying and evaluating novel artificial sweeteners. Two datasets, SweetnersDB and SweetpredDB, were identified to contain quantified sweetness data for a large set of molecules. Both were collected as part of separate studies into building a predictive model for relative sweetness. SweetnersDB contains 316 molecules and SweetpredDB contains 671. Of the 316 molecules in SweetnersDB, 300 of those are also covered in SweetpredDB (Chéron et al., 2017a), (Goel et al., 2023).

### 2.2 Odor and flavor

The following databases and datasets contain either odor or odor and taste. As described previously, flavor is a combination of taste, odor, and physical sensations within the mouth (Sánchez-Rodríguez et al. Yahia, 2019), (American Chemical Society, 2013), (ISO 5492). The key role that odor plays in flavor perception means that flavor and odor are inextricably linked. Large flavor databases such as FlavorDB and FooDB aggregate taste and odor information to create the flavor profile of the chemicals in their databases (Garg et al., 2018), (FooDB). The only exception to this is Fenaroli's Handbook of Flavor Ingredients, which has slightly different descriptors for some chemical's odor and flavor, based on what percepts are recognized when tasting or smelling a chemical at certain thresholds (Burdock, 2009). A challenge with odor data is the sheer number of labels used to describe different odors. Not all databases report the number of unique labels, but in several of the databases catalogued here it numbered in the hundreds. Odor labels are also for single chemicals only and do not consider how chemical interactions may change odor perception. Some chemicals also may be perceived differently at different concentrations, which is not taken into account by existing odor data resources (Gross-Isseroff and Lancet, 1988a).

**Pyrfume.** Pyrfume itself is not a database, but a Python library that curates and unifies a wide selection of the many disparate odor databases and datasets. The data in Pyrfume is in a consistent format and is designed to be quickly and easily ingested by machine learning platforms. Although Pyrfume contains over 40 datasets related to olfaction, only the 12 that are directly related to odor classification will be presented here. Some of the datasets included do not have perceptual labels but instead only mark whether a given chemical is an odorant or not, catalogue odor receptors, or have other odor related information. Such data, while potentially useful for olfaction-based machine learning tasks, is outside the scope of this review. Table 2 includes a column specifying whether each dataset is included in Pyrfume (Hamel et al., 2024).

**Perfume and flavor chemicals: (aroma chemicals), Vol 2.** Steffen Arctander's "Perfume and Flavor Chemicals" is a landmark reference book published in 1969 that provides a list of 3102 compounds used in the fragrance and flavor industries. Despite its age, the book remains an important resource for chemical/odor data (Arctander, 1969).

**Flavornet.** Despite having “flavor” in its name, Flavornet is a database of chemicals linked with odor descriptors. Flavornet is a catalog of 783 different chemical odorants, each classified as one or more of 197 different odors. Odor descriptors are derived from the ASTM D-66 categories of food odors. Data is derived from literature where quantitative GCO is used to detect odorants. When first published in 1998, Flavornet had 346 compounds, but has been updated as of 2004 to its current total (Arn and Acree, 1998).

**Fenaroli's Handbook of Flavor Ingredients.** Fenaroli's Handbook of Flavor Ingredients is a reference handbook that has been in print since 1971. The most recent publication is the 6th edition from 2009, and it contains 1107 compounds along with 1530 associated foods. The handbook is considered an industry standard and contains a wealth of information about each compound, including the threshold values for the odor and taste, a description of the aromatic profile, uses in food, and more. However, the handbook is designed for human use rather than to be easily ingested into a predictive model pipeline (Burdock, 2009).

**FlavorDB.** FlavorDB is a compilation of various molecular flavor resources into one consolidated database. FlavorDB contains 25,595 molecules and assigns a flavor, taste, and odor profile to each one based on various sources. Largely a compilation of existing molecular taste and odor resources, FlavorDB sources data from FooDB, Flavornet, SuperSweet, BitterDB, and Fenaroli's Handbook of Flavor Ingredients. A literature review was also performed to source additional molecular flavor pairings. Of those molecules, 2254 also have one or more associated foods. FlavorDB compiles 936 unique foods broken down into 34 categories. Food-chemical pairings are sourced from FooDB (Garg et al., 2018). In 2022, FlavorDB was updated to FlavorDB2. FlavorDB2 contains the same molecules, flavor profiles, and food pairings, but features updated metadata such as additional molecular identifiers, taste/odor threshold data, among others (Grover et al., 2022).

**SuperScent.** SuperScent contains 2147 molecules, each with structural data and one or more odor descriptors. The database can be searched by name, PubChem ID, or by structure. The data is from literature as well as existing databases, such as PubChem (Dunkel et al., 2009). At the time of writing, the database is no longer available at the URL specified in the original publication. However, the database OlfactionBase claims SuperScent as one of several sources for its data, so it can still be accessed albeit through another database (Sharma et al., 2021a).

**FooDB.** FooDB is a database that links 797 different foods with 70,926 chemical compounds. About 15,750 of these compounds have known links to foods, while 55,176 have expected or predicted links to foods. 2816 of these compounds also include a flavor profile. These flavor profiles aggregate taste and odor data derived from various other databases, including Flavornet and the Good Scents Company flavor and fragrance information catalog (FooDB).

**OlfactionBase.** OlfactionBase is a very comprehensive database of odorants, their associated odors, odor receptors, and odor receptor/odorant pairs. There are 3985 catalogued odorants as well as 1124 chemicals confirmed to be odorless. Moreover, odor percepts are grouped into primary odors and sub-odors. Odorant data is sourced from several large odor databases including AromaDB, Leffingwell's Flavor-Base, Flavornet, PubChem, Sigma Aldrich, SuperScent, and The Good Scents Company. Given that it encompasses so many large odor databases, OlfactionBase may be the most comprehensive odor database currently available.

**PungentDB.** PungentDB is a database of 205 compounds that exhibit pungency as part of their flavor profile. The database also includes 231 traditional Chinese medicine sources of these compounds. Data is sourced from literature as well as existing flavor databases such as FlavorDB and FooDB. Potential medicinal uses with references are also included with each compound (Chen et al., 2024a).

### 2.3 Texture

Texture data differs from taste and odor data in that it measures and quantifies the entire food based on one or more attributes, establishing a direct link between the food and its texture. In contrast, taste and odor data associate specific chemicals with tastes and or odors. The presence of that chemical in a food may or may not be known. Furthermore, techniques for analyzing food texture are inherently quantitative rather than categorical. Food texture data does not appear to be as prevalent as taste or odor data, but Texture Profile Analysis (TPA) is a popular method by which the food industry tests and compares textural properties of food. TPA is performed either with a sensory panel of human testers or by machine. Depending on the food being measured, different characteristics may or may not be applicable, hence the need for different parameters based on different food types, i.e. solid, semi-solid, and liquid (Szczesniak, 2002b). Although there is no comprehensive food texture database, some studies can be found where TPA is performed on a set of one specific food item or class of food items. Three such datasets are presented in Table 2.

## 3 Models

### 3.1 Taste prediction models

Much work has been done in the field of taste prediction. At first glance, the problem appears well-defined: given a substance, predict one or more taste labels usually based on the five basic tastes: sweet, salty, sour, bitter, and umami. However, there are important nuances to keep in mind.

The input data is often, but not always, in the form of chemical structures, so the first step is often interpreting this data. Various software exists to extract molecular descriptors and molecular fingerprints that can encode either the 2D or 3D structure of a molecule. Dragon, Mordred, RDKit, ChemoPy are a few examples of commonly used software for this purpose (Dragon 7), (Moriwaki et al., 2018), (RDKit open), (Cao et al., 2013). Different programs may extract different features, and some may perform better than others, depending on the given task and dataset. Various studies have compared different feature extractors and different combinations of features from different programs (Wang et al., 2021), (Bouysset et al., 2020), (Lee et al., 2022), (Banerjee and Preissner, 2018), (Tuwani et al., 2019), (Maroni et al., 2022), (Yang et al., 2022). The availability of these programs should also be taken into consideration. For example, some of these programs are paid, closed-source tools like Dragon, whereas others are free and open source like Mordred, RDKit, and ChemoPy. After the feature extraction step, there may be more molecular descriptors than there are molecules in a dataset, for example Dragon 7.0 boasts the capability of extracting 5270 descriptors, which is already larger than many molecular taste datasets (Dragon 7.0 - kode chemoinformatics). As such, feature selection becomes an important step in most taste prediction pipelines. Too many features in a model can lead to problems such as overfitting, the “curse of dimensionality,” and leads to challenges in model explainability (Trunk, 1979) (Ying, 2019).

An alternative to molecular descriptors and fingerprints is the use of graph neural nets (GNN). In this case, the structure of a molecule is encoded as a graph, where each atom is a node, and each bond is an edge. Node and edge features representing atomic features and bond features may be included as well. These features can be used to encode the atom types, bond types, and more (Sun et al., 2020), (Yang et al., 2019).

There have been several studies comparing the performance of descriptor-based models and graph-based models, and there is not yet a consensus on which is more effective in general. Several studies show that graph-based models outperform descriptor-based models (Yang et al., 2019), (Wu et al., 2018), (Korolev et al., 2020), (Withnall et al., 2020), (Hop et al., 2018), but others have shown descriptor models may still be superior, especially in terms of computational complexity (Jiang et al., 2021), (Mayr et al., 2018). The interpretability of these methods may also be taken into consideration. GNN's, like many deep learning methods, may be less interpretable than traditional ML methods. However, emerging research into GNN explainability may change this, and there have been several novel algorithms developed to help interpret GNNs (Wu et al., 2023a), (Wang et al., 2023), (Agarwal et al., 2023a).

As a newer framework for exploring the relationship between chemical structure and chemical properties, there are few examples of GNNs in taste prediction. However, a 2023 paper by Song et al. indicated that they may outperform other models in the domain of sweetness prediction. In that paper, Song et al. compared the performance of GNN-based models, fingerprint-based models, and CNN-based models for predicting sweetness, bitterness, and umaminess. They demonstrated that GNN-based models appear to outperform fingerprint and CNN-based models for sweet and bitter prediction. The best performing model tested was a consensus model incorporating both the GNN and fingerprint models, indicating that these types of chemical representations may have a complementary relationship (Song et al., 2023). This consensus model achieved an accuracy of 0.90 and an F1 score of 0.85 for sweet prediction. For bitter prediction, the results were similar with an accuracy of 0.90 and F1 score of 0.88.

However, superior results were achieved by Banjeree et al. using a model trained only on molecular fingerprint-based features. They trained random forests on binary molecular fingerprints to identify sweet and bitter chemicals. For predicting sweet molecules, the model achieved an accuracy of 0.97 and an F1 score of 0.92. In the bitter prediction task, the model's accuracy of 0.97 and F1 score was 0.95. (Banerjee and Preissner, 2018). More research is needed in evaluating the effectiveness of graph-based methods compared to molecular fingerprint-based methods.

Not all studies use molecular structure data, some focus on the amino acid sequences of various peptides. Peptides have been shown to play a significant role in the taste of certain foods. Many bitter and umami peptides have been identified, as well as a handful of sweet peptides. Some peptides have also been shown to enhance or suppress certain tastes (Iwaniak et al., 2016).

Various studies have shown that bitter and umami peptides can be predicted based on a given peptide's amino acid sequence (Charoenkwan et al., 2020b), (Charoenkwan et al., 2021a), (Charoenkwan et al., 2021b), (Charoenkwan et al., 2021c). In these cases, the preprocessing steps will of course be slightly different. In general, taste models that make predictions on peptides use the amino acid sequences of those peptides to extract features. One example is calculating amino acid and dipeptide propensity scores, as was done by Charoenkwan et al. in their work predicting umami peptides (Charoenkwan et al., 2020b). More recent peptide prediction models have leveraged transformer models such as BERT to operate directly on amino acid sequences (Charoenkwan et al., 2021a), (Zhang et al., 2023).

The models used in these predictions are diverse, but in general it seems traditional deep learning techniques such as random forests and support vector machines are slightly preferred over more complex deep learning methods. The relatively small size of many molecular-taste datasets available may make deep learning methods perform worse, given their propensity to overfitting on smaller datasets (Charilaou and Battat, 2022), (Janiesch et al., 2021).

Finally, the prediction goals of taste models are diverse as well. Although most studies simply classify chemicals into one or more taste groups, there are a growing number of models designed to quantify the sweetness of molecules (Goel et al., 2023), (Bouysset et al., 2020), (Yang et al., 2022), (Zheng et al., 2019), (Chéron et al., 2017b). So far, the only quantitative taste prediction models predict the relative sweetness of molecules. This is perhaps due to the general lack of quantitative taste data overall, as well as a growing interest in discovering novel artificial sweeteners (Chen et al., 2023). In the case of sweetness, it is quantified by “relative sweetness.” Relative sweetness is a measure used to compare the sweetness intensity of various sweeteners against sucrose (table sugar) as a standard reference. The relative sweetness of sucrose is assigned an arbitrary value of 100, and other sweeteners are ranked in comparison to this standard (Lichtenstein, 1948). In a recent paper, various sources of quantitative sweetness data were combined into a dataset of 671 molecules. Seven models were compared and a gradient boosting regressor was found to perform the best, achieving a correlation coefficient of 0.94 (Goel et al., 2023).

As shown previously, the amount of readily available labeled taste data is unbalanced. While there exists substantial data for sweet and bitter compounds, umami data is much sparser, with sour and salty data even less common still. It makes sense then that the prediction targets of the models in literature similarly follow this trend. Only one model was found that attempts to predict sour chemicals. In 2021, Fritz et al. describe three models, each achieving strong results in predicting sweet, bitter, and sour molecules. Each model uses a random forest as well as various oversampling techniques to overcome issues with class imbalance. The models for sweet, bitter and sour achieved accuracies of 0.89, 0.90, and 0.97 as well as F1 scores of 0.88, 0.88, and 0.84 respectively. Notably, the researchers were also able to predict which of 25 bitter taste receptors each identified bitter compound interacts with (Fritz et al., 2021).

Although Fritz et al. made three separate models for three different tastes, true multilabel predictive models have been explored as well. A recent paper from 2024 by Androutsos et al. outlines a multi-objective model for predicting bitter, sweet, umami, and “other” molecules. The “other” class of chemicals consists of molecules labeled as either non-sweet, tasteless, sour, or salty, none of which were large enough to justify their own class. Using molecular features generated by Mordred, they trained a random forest optimized via an evolutionary algorithm. Bitter and sweet were able to be predicted reliably, with an accuracy of 0.82 and 0.80 and an F1 score of 0.79 and 0.75, respectively. Prediction results for the “other” class were more limited, with an accuracy of 0.84 but an F1 score of only 0.43. Due to the small number of umami compounds documented in literature, they were unable to provide test data performance for umami classification, however it showed encouraging results in the 10-fold cross-validation training performance (Androutsos et al., 2024).

### 3.2 Odor prediction models

In general, odor prediction pipelines tend to look similar to taste prediction pipelines. Both tasks relate chemical structure to chemical properties, so odor prediction starts by extracting features from chemical structure data, much like taste prediction.

One interesting method by Sharma et al. for extracting structural information was to generate images of the molecules using the python library RDKit (RDKit open). These images display the structure of the molecule, the bond types, and encode different elements as distinct colors. The images were fed into a pre-trained CNN-based image classification model which outputs a prediction based on 104 odor percepts. The best performing model achieved an accuracy and F1 score of 0.98 and 0.88 respectively. Despite the difficulty of a 104-way classification problem, 103 of the odor percepts were able to be successfully predicted (Sharma et al., 2021b).

Odor prediction presents greater challenges than taste prediction due to its complex nature. Unlike taste, odors cannot be easily quantified or categorized. To illustrate this difference, consider how humans perceive color: three types of cone cells correspond roughly to our perception of red, green, and blue, allowing us to break down color space in terms of these three primary colors' intensities (Kremers et al., 2016). Similarly, taste can be analyzed based on receptors corresponding to the five basic tastes (Gravina et al., 2013). Olfaction, however, is an entirely different matter. Humans possess an estimated 400 different olfactory receptors, and recent research suggests we may be capable of distinguishing over one trillion distinct smells (Bushdid et al., 2014), (Rinaldi, 2007). This complexity is compounded by the inconsistent relationship between molecular structure and sensory perception. While it is generally accepted that molecular structure relates to a molecule's sensory characteristics, notable exceptions exist where structurally similar molecules produce different sensory experiences, and structurally dissimilar molecules elicit similar sensory responses (Sell, 2006).

Moreover, the lack of a standardized system for categorizing olfactory characteristics poses additional difficulties. Even well-established databases of chemical sensory information rely on subjective descriptions of odorants, highlighting the challenge of developing a universal framework for odor classification and prediction. As such, the body of research concerning molecular odor prediction is often centered around addressing these unique issues. For example, in a 2018 paper by Yuji Nozaki and Takamichi Nakamoto, researchers attempted to tackle the problem of a disparate labeling system of odorants by clustering odor descriptors using natural language processing techniques. Two clustering techniques were performed, the first measured the correlation coefficient between all pairs of odor descriptors. It was hypothesized that certain descriptors will often appear together if they describe a similar odor characteristic. For example, “rose” and “violet” both describe a floral aroma and thus are likely to appear together more frequently than “rose” and “sulfurous.” The second clustering technique encodes each descriptor as a vector and then clusters based on cosine similarity between word vectors. The second clustering technique leveraged Word2Vec, a natural language processing method developed by Mikolov et al. at Google that represents words as dense vector embeddings that capture semantic relationships between words based on their context within large text corpora (Mikolov et al.). Odor descriptors were encoded as vectors using Word2Vec and were clustered based on their similarity based on cosine distance. Mass spectra data of each molecule was used as input into a neural net that classified the molecule as one of k odor percept clusters. With a k set to 20, and using the Word2Vec clustering method, the authors reported a prediction accuracy of 53 % for true positives and 85 % for true negatives (Nozaki and Nakamoto, 2018).

Perhaps the most comprehensive attempt of mapping “odor space” can be found in the 2023 paper by Google Inc. “A principal odor map unifies diverse tasks in olfactory perception.” In the paper, researchers outline the architecture for an MPNN that both classifies odorant molecules as well as organizes them in a semantically meaningful space. That is, two odorants that have similar perceptual qualities will be near one another in the generated odor map. The predictive power of the model was compared to a panel of human testers, and it was found that the model generally predicted better than the median human panelist. The predictive success of the model indicates that the embeddings generated by the model may indeed capture relevant sensory information, and this was confirmed when the embeddings were used to train linear models with similar predictive strength (Lee et al., 2023).

### 3.3 Texture prediction models

Although consumers generally place significant importance on a food's texture, relatively few studies have focused on texture prediction (Szczesniak and Kahn, 1971a). Although TPA is an established and effective way of analyzing food texture, it is a destructive process and may be too time consuming for certain applications, such as integration into a quality assurance pipeline. As a result, many texture prediction methods involve rapid and non-destructive data collection, such as hyperspectral imagery or near-infrared reflectance spectroscopy (He et al., 2014), (Zhou et al., 2020), (Liu et al., 2003), (Ma et al., 2017). One limitation seen in many of these predictive models is a lack of data. Even the largest dataset identified only had 387 samples. Of the nine food texture prediction studies identified, four used datasets with less than 100 total samples. One reason for this may be the costly and time-consuming process of texture data collection. Of the texture studies identified, all except one collected original data. (Table 3a).

**Table 3a:** Model descriptions.

Name	Type	Data source	Data quantity	Goal	Model	Year
SMILES to Smell: Decoding the Structure–Odor Relationship of Chemical Compounds Using the Deep Neural Network Approach (Sharma et al., 2021b)	Odor	PubChem, GoodScents, Sigma-Aldrich, FlavorBase, Flavornet, SuperScent, ODORactor, AromaDB	5185 chems	104 odors	RF, DNN∗ (PPMF)/CNN∗ (images)	2021
Predicting odor from vibrational spectra: a data-driven approach (Ameta et al., 2024)	Odor	Leffingwell, Chemistry Webbook	3018 chems	109 odors	MLP	2024
Predicting natural language descriptions of mono-molecular odorants (Gutiérrez et al., 2018)	Odor	DREAM, Dravnieks, Dragon molecular descriptors	476 chems, 19 odors	131 odors	Elastic net regression	2018
A principal odor map unifies diverse tasks in olfactory perception (Lee et al., 2023)	Odor	GoodScents, Leffingwell	∼5000 chems, 400 odors	138 odors	MPNN	2023
Odor Impression Prediction from Mass Spectra (Nozaki and Nakamoto, 2016)	Odor	Chemistry WebBook, Dravnieks	121 chems, 144 odors	144 odors	Autoencoder for dimensionality reduction, then 9-layer NN	2016
Application of artificial intelligence to decode the relationships between smell, olfactory receptors and small molecules (Achebouche et al., 2022)	Odor	GS/LF	5955 chem, 160 odors, 106 OR's	160 odors/23 odor groups	CNN, GCN∗	2022
XGBoost odor prediction model: finding the structure-odor relationship of odorant molecules using the extreme gradient boosting algorithm (Tyagi et al., 2024)	Odor	OlfactionBase	1278 chems	7 odors	XGBoost	2023
Predictive modeling for odor character of a chemical using machine learning combined with natural language processing (Nozaki and Nakamoto, 2018)	Odor	Chemistry Webbook, Sigma-Aldrich	999 chems, 138 odors	Odor groups of size 6, 20, and 30	Autoencoder for dimensionality reduction, then 4-layer NN	2018
iBitter-SCM: Identification and characterization of bitter peptides using a scoring card method with propensity scores of dipeptides (Charoenkwan et al., 2020a)	Taste	BIOPEP, literature review	640 peptides	Bitter	Scoring card method	2020
e-Bitter: Bitterant Prediction by the Consensus Voting From the Machine-Learning Methods (Zheng et al., 2018)	Taste	BitterDB, SuperSweet, SweetenersDB, literature review	1299 chems	Bitter	Ensemble: KNN, SVM, RF, GBM, and DNN	2018
BitterX: a tool for understanding bitter taste in humans (Huang et al., 2016)	Taste	BitterDB, literature review	1078 chems	Bitter	SVM + GA	2016
Bitter or not? BitterPredict, a tool for predicting taste from chemical structure (Dagan-Wiener et al., 2017)	Taste	BitterDB, TOXNET, literature review	2608 chems	Bitter	AdaBoost	2017
BERT4Bitter: a bidirectional encoder representations from transformers (BERT)-based model for improving the prediction of bitter peptides (Charoenkwan et al., 2021a)	Taste	BTP640	640 peptides	Bitter/non-bitter	BERT∗, CNN, LSTM	2021
iBitter-Fuse: A Novel Sequence-Based Bitter Peptide Predictor by Fusing Multi-View Features (Charoenkwan et al., 2021c)	Taste	BTP640	640 peptides	Bitter/non-bitter	SVM	2021
Prediction of bitterant and sweetener using structure-taste relationship models based on an artificial neural network (Bo et al., 2022)	Taste	BitterDB, SuperSweet, FlavorDB	4599 chems	Bitter/non-bitter, sweet/non-sweet, and bitter/sweet	CNN, MLP∗	2022
Novel scaffold of natural compound eliciting sweet taste revealed by machine learning (Bouysset et al., 2020)	Taste	SweetenersDB	316 chems	Relative sweetness	RF, SVM, AdaBoost∗, k-NN	2020
Sweetness prediction of natural compounds (Chéron et al., 2017b)	Taste	SweetenersDB	316 chems	Relative sweetness	SVR∗, RF	2017
A QSTR-Based Expert System to Predict Sweetness of Molecules (Rojas et al., 2017)	Taste	TastesDB	649 chems	Sweet	k-NN + PLSDA consensus	2017
Machine learning models to predict sweetness of molecules (Goel et al., 2023)	Taste	SweetpredDB	671 chems	Relative sweetness	Gradient Boost Regressor∗, RF	2023
VirtualTaste: a web server for the prediction of organoleptic properties of chemical compounds (Fritz et al., 2021)	Taste	SuperSweet, BitterDB, ChEMBL, literature review	4111 chems	Sweet, bitter, sour	RF	2021
Predicting multiple taste sensations with a multiobjective machine learning method (Androutsos et al., 2024)	Taste	BitterDB Fenaroli SuperSweet SweetenersDB UMP442 ChemTastesDB BIOPEP-UWM, literature review	4717 chems	Sweet, bitter, umami, other	RF	2024
BitterSweetForest: A Random Forest Based Binary Classifier to Predict Bitterness and Sweetness of Chemical Compounds (Banerjee and Preissner, 2018)	Taste	SuperSweet, BitterDB	1202 chems	Sweet/bitter	RF	2018
Informed classification of sweeteners/bitterants compounds via explainable machine learning (Maroni et al., 2022)	Taste	BitterDB, Fenaroli, SuperSweet, GoodScents, SweetenersDB, literature review	2686 chems	Sweet/bitter	Logistic Regression, k-NN, RF, LightGBM∗, MLP	2022
BitterSweet: Building machine learning models for predicting the bitter and sweet taste of small molecules (Tuwani et al., 2019)	Taste	BitterDB, Fenaroli, SuperSweet, SweetenersDB, literature review	4804 chems	Sweet/non-sweet bitter/non-bitter	RF∗ (bitter), Ridge Logistic Regression, AdaBoost∗ (Sweet)	2019
e-Sweet: A Machine-Learning Based Platform for the Prediction of Sweetener and Its Relative Sweetness (Zheng et al., 2019)	Taste	SuperSweet, SweetenersDB, literature review	1380 chems	Sweet/non-sweet, relative sweetness	KNN, SVM, GBM, RF, and DNN consensus model	2019
A Comprehensive Comparative Analysis of Deep Learning Based Feature Representations for Molecular Taste Prediction (Song et al., 2023)	Taste	ChemTastesDB	2601 chems	Sweet/non-sweet, bitter/non-bitter, umami/non-umami	GNN, CNN, consensus model∗	2023
BoostSweet: Learning molecular perceptual representations of sweeteners (Lee et al., 2022)	Taste	BitterSweet	2291 chems	Sweet/non-sweet	RF, XGB, LGBM, FCN soft vote ensemble	2022
A novel multi-layer prediction approach for sweetness evaluation based on systematic machine learning modeling (Yang et al., 2022)	Taste	Taste DB and LogSw DB	2861 chems	Relative sweetness	Decision Tree, kNN, SVM, RF, XGBoost, GBT, PLS, SVR∗	2022
Toward a general and interpretable umami taste predictor using a multi-objective machine learning approach (Pallante et al., 2022)	Taste	UMP442	442 peptides	Umami	SVM Ensemble	2022
iUmami-SCM: A Novel Sequence-Based Predictor for Prediction and Analysis of Umami Peptides Using a Scoring Card Method with Propensity Scores of Dipeptides (Charoenkwan et al., 2020b)	Taste	BIOPEP-UWM, literature review	442 peptides	Umami	Scoring Card Method	2020
UMPred-FRL: A New Approach for Accurate Prediction of Umami Peptides Using Feature Representation Learning (Charoenkwan et al., 2021b)	Taste	UMP442	442 peptides	Umami	SVM	2021
Umami-BERT: An interpretable BERT-based model for umami peptides prediction (Zhang et al., 2023)	Taste	UMP789	789 peptides	Umami	BERT	2023
Umami-MRNN: Deep learning-based prediction of umami peptide using RNN and MLP (Qi et al., 2023)	Taste	BIOPEP-UWM, literature review	499 peptides	Umami	MLP + RNN	2023
Artificial neural networks modeling of non-fat yogurt texture properties: effect of process conditions and food composition (Batista et al., 2021)	Texture	Self-collected	13 samples	Firmness, cohesiveness, adhesiveness, and gumminess	ANN	2021
Predicting the Textural Properties of Plant-Based Meat Analogs with Machine Learning (Kircali Ata et al., 2023)	Texture	Literature review	54 samples	Hardness, chewiness	Ridge∗, RF, XGBoost, KNN∗, MLP	2023
Rapid and Non-Invasive Assessment of Texture Profile Analysis of Common Carp (Cyprinus carpio L.) Using Hyperspectral Imaging and Machine Learning (Cao et al., 2023)	Texture	Self-collected	387 samples	Gumminess, springiness, cohesiveness, resilience, hardness, brittleness, adhesiveness, chewiness	ANN, SVM, and PLSR (different models optimal for different objectives)	2023
Prediction of textural characteristics in low-fat mozzarella cheese by Hyperspectral imaging using machine learning methods (Jahani et al., 2024)	Texture	Self-collected	36 samples	Hardness, adhesiveness, springiness, cohesiveness, gumminess, chewiness	MLR, PLSR, SVM, MLP∗, RF∗, consensus model∗	2024
Non-destructive prediction of texture of frozen/thaw raw beef by Raman spectroscopy (Chen et al., 2020)	Texture	Self-collected	16 samples	Tenderness, chewiness, firmness, hardness, springiness	PLSR	2020
Prediction of textural changes in grass carp fillets as affected by vacuum freeze drying using hyperspectral imaging based on integrated group wavelengths (Ma et al., 2017)	Texture	Self-collected	112 samples	WBSF, hardness, gumminess, chewiness	PLSR	2017
Prediction of color, texture, and sensory characteristics of beef steaks by visible and near infrared reflectance spectroscopy. A feasibility study (Liu et al., 2003)	Texture	Self-collected	113 samples	Tender/tough	SIMCA∗, PLSR	2003
Rapid determination of the textural properties of silver carp (Hypophthalmichthys molitrix) using near-infrared reflectance spectroscopy and chemometrics (Zhou et al., 2020)	Texture	Self-collected	150 samples	WHC, hardness, resilience, springiness, chewiness, shear force	PLSR	2020
Potential of hyperspectral imaging combined with chemometric analysis for assessing and visualising tenderness distribution in raw farmed salmon fillets (He et al., 2014)	Texture	Self-collected	164 samples	WBSF	PLSR, SVM∗	2014

A list of taste, odor, and texture predicting models with relevant information such as the type of model used, publication date, and data sources.

**Table 3b:** Model results.

Name	Acc	NER	F1	Pr	ROC AUC	PR AUC	R	MCC	Sn	Sp	R^2^	RMSE
SMILES to Smell: Decoding the Structure–Odor Relationship of Chemical Compounds Using the Deep Neural Network Approach (Sharma et al., 2021b)	0.98	–	0.88	0.97	micro avg: 0.87 macro avg: 0.7	micro avg: 0.74 macro avg: 0.75	–	–	0.84	–	–	–
Predicting odor from vibrational spectra: a data-driven approach (Ameta et al., 2024)	–	–	0.41	0.36	–	–	–	–	0.47	–	–	–
Predicting natural language descriptions of mono-molecular odorants (Gutiérrez et al., 2018)	–	–	–	–	0.66	–	–	–	–	–	–	–
A principal odor map unifies diverse tasks in olfactory perception (Lee et al., 2023)	–	–	–	–	0.89∗	–	–	–	–	–	–	–
Odor Impression Prediction from Mass Spectra (Nozaki and Nakamoto, 2016)	–	–	–	–	–	–	0.76∗	–	–	–	–	–
Application of artificial intelligence to decode the relationships between smell, olfactory receptors and small molecules (Achebouche et al., 2022)												
*odor note:*	–	–	–	–	0.82∗	0.24∗	–	–	–	–	–	–
*odor group:*	–	–	–	–	0.80∗	0.40∗	–	–	–	–	–	–
XGBoost odor prediction model: finding the structure-odor relationship of odorant molecules using the extreme gradient boosting algorithm (Tyagi et al., 2024)	0.99	–	0.99	0.99	0.99	0.99	–	0.99	0.99	–	–	–
Predictive modeling for odor character of a chemical using machine learning combined with natural language processing (Nozaki and Nakamoto, 2018)	0.69∗	–	0.63∗	0.78∗	–	–	–	0.40∗	0.53∗	0.85∗	–	–
iBitter-SCM: Identification and characterization of bitter peptides using a scoring card method with propensity scores of dipeptides (Charoenkwan et al., 2020a)	0.84	–	–	–	0.90	–	–	0.69	0.84	0.84	–	–
e-Bitter: Bitterant Prediction by the Consensus Voting From the Machine-Learning Methods (Zheng et al., 2018)	0.93	–	0.94	0.92	–	–	–	0.86	0.95	0.90	–	–
BitterX: a tool for understanding bitter taste in humans (Huang et al., 2016)	0.92	–	–	0.91	0.96	–	–	–	0.94	0.91	–	–
Bitter or not? BitterPredict, a tool for predicting taste from chemical structure (Dagan-Wiener et al., 2017)	0.83	–	–	–	–	–	–	–	0.77	0.86	–	–
BERT4Bitter: a bidirectional encoder representations from transformers (BERT)-based model for improving the prediction of bitter peptides (Charoenkwan et al., 2021a)	0.92	–	–	–	0.96	–	–	0.84	0.94	0.91	–	–
iBitter-Fuse: A Novel Sequence-Based Bitter Peptide Predictor by Fusing Multi-View Features (Charoenkwan et al., 2021c)	0.93	–	–	–	0.93	–	–	0.86	0.94	0.92	–	–
Prediction of bitterant and sweetener using structure-taste relationship models based on an artificial neural network (Bo et al., 2022)	0.88	–	–	0.89	0.95	–	–	0.75	0.83	0.93	–	–
Novel scaffold of natural compound eliciting sweet taste revealed by machine learning (Bouysset et al., 2020)	–	–	–	–	–	–	–	–	–	–	0.75	–
Sweetness prediction of natural compounds (Chéron et al., 2017b)	–	–	–	–	–	–	–	–	–	–	0.85	–
A QSTR-Based Expert System to Predict Sweetness of Molecules (Rojas et al., 2017)	–	0.85	–	–	–	–	–	–	0.88	0.82	–	–
Machine learning models to predict sweetness of molecules (Goel et al., 2023)	–	–	–	–	–	–	0.94	–	–	–	–	0.33
VirtualTaste: a web server for the prediction of organoleptic properties of chemical compounds (Fritz et al., 2021)												
*sweet:*	0.89	–	0.88	–	0.95	–	–	–	0.68	0.92	–	–
*bitter:*	0.90	–	0.88	–	0.96	–	–	–	0.88	0.97	–	–
*sour:*	0.97	–	0.84	–	0.99	–	–	–	0.80	0.99	–	–
Predicting multiple taste sensations with a multiobjective machine learning method (Androutsos et al., 2024)	0.72	–	0.74	0.79	0.87	–	–	–	0.72	–	–	–
BitterSweetForest: A Random Forest Based Binary Classifier to Predict Bitterness and Sweetness of Chemical Compounds (Banerjee and Preissner, 2018)												
*sweet*	0.97	–	0.92	–	0.98	–	–	–	0.91	0.97	–	–
*bitter*	0.97	–	0.95	–	0.98	–	–	–	0.97	0.91	–	–
Informed classification of sweeteners/bitterants compounds via explainable machine learning (Maroni et al., 2022)	–	–	0.88∗	0.87∗	0.95∗	0.94∗	–	–	0.89∗	–	–	–
BitterSweet: Building machine learning models for predicting the bitter and sweet taste of small molecules (Tuwani et al., 2019)												
*sweet*	–	0.83	0.86	–	0.88	0.95	–	–	0.79	0.88	–	–
*bitter*	–	0.82	0.86	–	0.88	0.93	–	–	0.85	0.79	–	–
e-Sweet: A Machine-Learning Based Platform for the Prediction of Sweetener and Its Relative Sweetness (Zheng et al., 2019)	0.91	0.9	0.88	0.9	–	–	–	0.81	0.86	0.94	0.78	0.28
A Comprehensive Comparative Analysis of Deep Learning Based Feature Representations for Molecular Taste Prediction (Song et al., 2023)												
*sweet*	0.90	–	0.85	0.84	–	–	–	–	0.86	0.92	–	–
*bitter*	0.89	–	0.87	0.93	–	–	–	–	0.82	0.95	–	–
*umami*	0.99	–	0.91	1.00	–	–	–	–	0.84	1.00	–	–
BoostSweet: Learning molecular perceptual representations of sweeteners (Lee et al., 2022)	–	0.90	0.91	–	0.96	0.97	–	–	0.91	0.90	–	–
A novel multi-layer prediction approach for sweetness evaluation based on systematic machine learning modeling (Yang et al., 2022)	–	–	–	–	–	–	–	–	–	–	0.85	0.48
Toward a general and interpretable umami taste predictor using a multi-objective machine learning approach (Pallante et al., 2022)	0.88	–	0.79	–	0.85	–	–	–	0.79	0.92	–	–
iUmami-SCM: A Novel Sequence-Based Predictor for Prediction and Analysis of Umami Peptides Using a Scoring Card Method with Propensity Scores of Dipeptides (Charoenkwan et al., 2020b)	0.87	–	–	–	0.90	–	–	0.68	0.71	0.93	–	–
UMPred-FRL: A New Approach for Accurate Prediction of Umami Peptides Using Feature Representation Learning (Charoenkwan et al., 2021b)	0.82	–	–	–	0.91	–	–	0.56	0.57	0.93	–	–
Umami-BERT: An interpretable BERT-based model for umami peptides prediction (Zhang et al., 2023)	0.95∗	–	–	–	0.97∗	–	–	0.85∗	0.97∗	1.00∗	–	–
Umami-MRNN: Deep learning-based prediction of umami peptide using RNN and MLP (Qi et al., 2023)	0.93	–	–	–	0.97	–	–	0.86	0.97	0.91	–	–
Artificial neural networks modeling of non-fat yogurt texture properties: effect of process conditions and food composition (Batista et al., 2021)												
*firmness*	–	–	–	–	–	–	–	–	–	–	1.00∗∗	0.00∗∗
*cohesiveness*	–	–	–	–	–	–	–	–	–	–	1.00∗∗	0.00∗∗
*adhesiveness*	–	–	–	–	–	–	–	–	–	–	0.98∗∗	0.00∗∗
*gumminess*	–	–	–	–	–	–	–	–	–	–	1.00∗∗	0.00∗∗
Predicting the Textural Properties of Plant-Based Meat Analogs with Machine Learning (Kircali Ata et al., 2023)												
*hardness*	–	–	–	–	–	–	–	–	–	–	–	10.10∗∗
*chewiness*	–	–	–	–	–	–	–	–	–	–	–	6.04∗∗
Rapid and Non-Invasive Assessment of Texture Profile Analysis of Common Carp (Cyprinus carpio L.) Using Hyperspectral Imaging and Machine Learning (Cao et al., 2023)	32 targets, see paper for full results.											
Prediction of textural characteristics in low-fat mozzarella cheese by Hyperspectral imaging using machine learning methods (Jahani et al., 2024)												
*hardness*	–	–	–	–	–	–	–	–	–	–	0.88∗∗	–
*adhesiveness*	–	–	–	–	–	–	–	–	–	–	0.81∗∗	–
*springiness*	–	–	–	–	–	–	–	–	–	–	0.85∗∗	–
*cohesiveness*	–	–	–	–	–	–	–	–	–	–	0.70∗∗	–
*gumminess*	–	–	–	–	–	–	–	–	–	–	0.82∗∗	–
*chewiness*	–	–	–	–	–	–	–	–	–	–	0.84∗∗	–
Non-destructive prediction of texture of frozen/thaw raw beef by Raman spectroscopy (Chen et al., 2020)												
*tenderness*	–	–	–	–	–	–	–	–	–	–	0.81∗∗	–
*chewiness*	–	–	–	–	–	–	–	–	–	–	0.80∗∗	–
*firmness*	–	–	–	–	–	–	–	–	–	–	0.81∗∗	–
*hardness*	–	–	–	–	–	–	–	–	–	–	0.82∗∗	–
*springiness*	–	–	–	–	–	–	–	–	–	–	0.53∗∗	–
Prediction of textural changes in grass carp fillets as affected by vacuum freeze drying using hyperspectral imaging based on integrated group wavelengths (Ma et al., 2017)												
*WBSF*	–	–	–	–	–	–	–	–	–	–	0.91∗∗	–
*hardness*	–	–	–	–	–	–	–	–	–	–	0.90∗∗	–
*gumminess*	–	–	–	–	–	–	–	–	–	–	0.88∗	–
*chewiness*	–	–	–	–	–	–	–	–	–	–	0.90∗∗	–
Prediction of color, texture, and sensory characteristics of beef steaks by visible and near infrared reflectance spectroscopy. A feasibility study (Liu et al., 2003)	0.96∗∗	–	–	–	–	–	–	–	–	–	–	–
Rapid determination of the textural properties of silver carp (Hypophthalmichthys molitrix) using near-infrared reflectance spectroscopy and chemometrics (Zhou et al., 2020)												
*WHC*	–	–	–	–	–	–	–	–	–	–	0.95∗∗	–
*hardness*	–	–	–	–	–	–	–	–	–	–	0.89∗∗	–
*resilience*	–	–	–	–	–	–	–	–	–	–	0.87∗∗	–
*springiness*	–	–	–	–	–	–	–	–	–	–	0.83∗∗	–
*chewiness*	–	–	–	–	–	–	–	–	–	–	0.92∗∗	–
*shear force*	–	–	–	–	–	–	–	–	–	–	0.88∗∗	–
Potential of hyperspectral imaging combined with chemometric analysis for assessing and visualising tenderness distribution in raw farmed salmon fillets (He et al., 2014)												
*WBSF*	–	–	–	–	–	–	–	–	–	–	0.90	–

The performance of all models listed in Table 3a. Metrics include accuracy (Acc), non-error rate (NER), F1 score, precision (Pr), area under the receiver operating characteristic curve (ROC AUC), R score, Matthews correlation coefficient (MCC), sensitivity (Sn), specificity (Sp), R^2^ score, and root mean squared error (RMSE).

Once data is collected, texture prediction usually involves predicting one or more texture characteristics. These characteristics are quantifiable and so regression models are commonly used. Characteristics include but are not limited to the Warner–Bratzler shear force (WBSF), a measure of tenderness in meats (Boccard et al., 1981), Water Holding Capacity (WHC), another important textural measurement of meats (Huff-Lonergan and Lonergan, 2005), as well as a list of other properties originally defined in 1963 by Brandt et al. (1963). These properties are typically predicted using traditional machine learning methods, with partial least squares regression (PLSR) being a common choice, seen in seven of the nine papers curated for this review.

## 4 Applications

Machine learning applications in chemical taste and odor prediction span a wide range of industries and scientific fields, with their specific use depending on whether taste, odor, or overall flavor profile is being predicted.

**Pest management.** One unique and impactful application has been in the discovery of novel mosquito repellants (Wei et al., 2022). The Centers for Disease Control and Prevention (CDC) considers mosquitos the "world's deadliest animal" due to their role in transmitting various deadly diseases, including malaria, which claimed approximately 608,000 lives in 2022 (World malaria report 2023), (CDC). Researchers have made significant strides in this area by training Graph Neural Networks (GNNs) on a database of about 19,000 chemicals, including known mosquito repellants. This approach successfully identified over 10 novel repellant chemicals that were at least as strong as current commercial repellants (Wei et al., 2022).

**Food and Beverage.** In the food and beverage industry, machine learning models for flavor prediction have the potential to enhance the production pipeline. Development, quality control, and personalized nutrition can all be targeted for improvement. These models can predict consumer preferences for new flavor combinations, optimize recipes by suggesting ingredient modifications, and reduce the time and cost associated with traditional sensory panels. They also play a crucial role in quality control by predicting flavor profiles based on ingredient composition and processing conditions, ensuring consistency across batches.

**Health and nutrition.** Accurate flavor prediction could play a key role in identifying novel ingredient substitutions that enhance a product's nutritional profile while preserving or even improving its original flavor. In particular, for consumers with certain conditions such as celiac disease or irritable bowel syndrome, restrictive diets have been linked with nutritional deficiencies (Staudacher et al., 2014), (Vici et al., 2016). Flavor prediction models could be used to provide additional options for individuals with conditions like these.

**Fragrance and cosmetics.** The fragrance and cosmetics industry can also benefit significantly from odor prediction models. In perfume formulation, these models can predict how different scent molecules will interact to create complex fragrances and suggest novel combinations of ingredients for unique scent profiles.

**Pharmaceuticals.** In the pharmaceutical sector, flavor prediction has important applications in improving medication palatability and masking bitter tastes. Machine learning models can predict and optimize the taste of oral medications, especially for pediatric formulations, enhancing patient compliance without compromising efficacy. Bitter taste in medicine has been linked to a lack of patient adherence, especially in children (Visciano and Schirone, 2020). It is also possible to identify compounds that effectively mask the bitter taste of certain drugs and predict the effectiveness of various taste-masking strategies (Margulis et al., 2021). Machine prediction of bitterants is important as human trials can be problematic due to the toxicity of many bitter compounds. (Bahia et al., 2018).

**Environmental monitoring and safety.** Beyond consumer products, taste and odor prediction have found applications in environmental monitoring and safety. These technologies are being used to predict the presence of harmful or toxic chemicals based on odor profiles, developing "electronic noses" for continuous air quality monitoring in industrial or urban settings (Capelli et al., 2014). In food safety, they can predict the presence of spoilage or contamination based on changes in taste or odor profiles, enabling the development of faster and less invasive methods for food quality assessment (Bax et al., 2020). As machine learning techniques continue to advance and more comprehensive datasets become available, the applications of flavor prediction are likely to expand further.

### 4.1 Limitations

There is still work to be done after identifying potentially useful taste, odor, and flavor chemicals. Bringing novel flavoring chemicals to market is not without its challenges. First, the safety and environmental impacts of ought to be carefully assessed. Within the food industry specifically, novel flavoring chemicals are subject to greater restrictions and a more rigorous approval process according to both US and EU food regulations, unless said chemical is already in widespread use (Flavourings), (Determining the Regulatory Status of a Food Ingredient, 2024). Many advanced machine learning models, particularly deep learning approaches, function as "black boxes." This lack of interpretability can be a barrier in critical applications like pharmaceuticals or food safety, where understanding the basis of a prediction is crucial for trust and validation.

## 5 Gap analysis

**Saltiness prediction.** There currently appears to be little research into predicting the saltiness of molecules. Although table salt (NaCl) is cheap and widely available, various studies have linked excess sodium consumption with several health conditions and diseases, including high blood pressure and cardiovascular disease (Morrison and Ness, 2011), (Mente et al., 2021), (Poggio et al., 2015). Furthermore, in studies comparing long term usage of the common salt substitute potassium chloride, researchers found a reduction in strokes, major cardiovascular events, and death while observing no serious adverse side effects in otherwise healthy patients (Tsai et al., 2022), (Neal et al., 2021). However, potassium chloride may not be suitable for individuals with certain health conditions that prevent the excretion of potassium, such as chronic kidney disease, diabetes mellitus, or those on certain medications (Greer et al., 2020). Within the databases compiled in this review, only eight compounds were identified as having a salty taste. Seven were from PlantMolecularTasteDB, and one from FlavorDB. The lack of data makes salty molecule prediction difficult. Future research should examine data augmentation strategies as well as the curation of additional salty compounds from literature. Recent research has also identified certain peptides as having the effect of enhancing perceived saltiness (Chen et al., 2021), (Chen et al., 2024b). Given the effectiveness of existing machine learning techniques for identifying the taste properties of peptides (Charoenkwan et al., 2021c), (Zhang et al., 2023), (Pallante et al., 2022), it follows that similar techniques might be well suited at detecting saltiness enhancing peptides.

**Building taste receptor models.** Further work is needed in incorporating taste receptors as part of taste prediction models. Taste receptors are not just found on the tongue and in fact play a wide variety of roles on various different systems in the body, being found in the heart, brain, and pancreas, and other areas (Kyriazis et al., 2014), (Foster et al., 2014), (Shaw et al., 2018), (Functionally expressed bitter taste receptor), (Deckmann, 2014). A better understanding of how different chemicals interact with these receptors could improve existing predictive models as well as answer other key questions about the nature of taste receptors within different organ systems. Taste is a sensation that involves both a chemical and a taste receptor, but all taste prediction models identified currently only focus on the structure of the tastants, rather than the receptors themselves. Recent advances in protein interaction prediction such as AphaFold 3 could be used to create superior prediction models (Abramson, 2024). Furthermore, a deeper understanding of taste receptor-chemical interactions could lead to advances in our understanding of how these receptors function in different organ systems.

**Mapping odor space.** The problem of quantifying the sensory characteristics of odors, and therefore flavors, is still unsolved. While the Principal Odor Map is one of the more comprehensive attempts at mapping out this sensory space, it has several shortcomings. It is limited to predicting the odor of single molecules and does not consider how sensory information changes with different chemical interactions, which may be more applicable to real world scenarios. Additionally, human odor space is theorized to be much larger than what the Principal Odor Map currently encompasses. Mapping odor space is still an open problem, and more research is needed. Future work could incorporate known olfactory receptor information. Another challenge in this area is to ensure that data is high quality. Some of the odor datasets that are commonly used in odor prediction models were compiled decades ago, and so there is no way to ensure the quality of their content. It has been shown that small impurities in a sample can have a major effect on odor perception (Paoli et al., 2017). Given that many odor prediction studies focus on single chemical-odor pairs, this presents a problem for both accurate prediction and accurate explanations of those predictions, as there is no way to guarantee that the original samples contained no impurities. As such data appears scarce within literature, additional experimental data may need to be collected in order build more robust odor sets that take into account factors such as chemical concentration and chemical interactions.

**Mapping texture space.** Each of the different texture parameters measured by TPA has a list of standard reference foods that correspond to each score of a given texture parameter. For example, the standard reference foods for hardness range from cream cheese which has a 1, and rock candy which has a 9. It would be beneficial to create a database of foods with texture scores calculated using TPA. Such a food texture database could be linked to food composition data sources, enabling future insights into the relationship between food texture and composition.

**Explainability in AI flavor models.** Further work in the explainability of taste and odor prediction models is needed. Explainability in AI has become an important topic, with various methods available to help explain traditionally “black-box” prediction models (Lundberg and Lee), (S. and S., 2023). Interpretable predictions are important for establishing trust in a model's output, and trust is essential in a field like flavor prediction where often the goal is to identify new compounds to be used in food production. Furthermore, the insights gained from why a model makes certain predictions can be used to inform future model development (Jiménez-Luna et al., 2020). Novel methods have been explored that are tailored to molecular property prediction, as well as benchmarks for evaluating the interpretability of a given model (Matveieva and Polishchuk, 2021). Graph neural nets are traditionally a black-box model, various attempts at explaining their predictions have been proposed and show promise at highlighting which graph nodes and edges are relevant to a given prediction (Agarwal et al., 2023b). Explainability of this type of model can yield insights into the specific atomic features, structural features, and substructural features of a molecule that lead to a given prediction (Wu et al., 2023b).

## 6 Discussion

A large-scale food texture database relating texture and food would be transformative in food texture prediction. Currently, food texture data is difficult to find and even in studies where food texture prediction is the goal, the underlying data is not always made publicly available. There exist standardized methods of obtaining reliable, quantifiable texture data through either human sensory panels or instrumental methods such as TPA. Ideally, a comprehensive food texture dataset would be enriched with complementary data, such as detailed food composition analysis or spectral imaging, to allow for interesting and useful texture predictions.

As shown previously, there are various databases relating chemical compounds with sensory information. However, there is not yet a database connecting actual foods to sensory data. We eat food, not chemicals, so while molecular taste and odor prediction can inform predictions made about food, there is currently no baseline to verify if such predictions apply to whole food items. Food sensory data exists in literature and is usually obtained by conducting sensory panels on trained experts, consumers, or both. A central repository for such data would provide researchers with a wealth of information that could be used to benchmark food sensory predictions as well as potentially elucidate the complex interactions among different taste and odor chemicals.

There are three key areas where future odor databases could make meaningful contributions in advancing odor research. Despite the existence of standardized methods for quantifying odors, the vast majority of odor data available are not quantified. Thus, it is not clear which odorants will have a greater effect on the overall odor profile of a food. Secondly, the available data often does not include concentration information. Concentration data is critical as the same chemical compound at different levels of concentration can elicit a different odor perception (Gross-Isseroff and Lancet, 1988b), (Laing et al., 2003). Finally, the interactions between compounds should also be considered. It has been shown that the combination of odors is not necessarily as simple as the sum of the combination's parts (Xu et al., 2020). Although it is not feasible to test and catalogue every possible odor combination, a large enough dataset of known combinations might allow for the training of an odor combination predictor.

## 7 Conclusion

Flavor and texture are fundamental properties of any product that is on the market, particularly when it comes to food and beverage. Being able to accurately predict their values is central to any R&D and manufacturing process. For example, in the case of food consumer packaged goods (CPG), a multi-trillion-dollar industry, consumer acceptance of any product is mainly driven by these two attributes (Szczesniak and Kahn, 1971b), (Drewnowski, 1997). Exploring the vast number of possibilities when mixing and processing formulations is inefficient, given the time it takes to create those samples, and the vast combinatorial space of the various formulations possible. Computationally identified flavor compounds and predictive models offer significant potential to streamline the research and development process in the food industry. These models can rapidly identify novel and targeted ingredient substitutions, which can lead to the creation of healthier products that maintain the flavor profile of the original. Beyond just food design, texture and odor prediction can be leveraged for food quality assurance. Instruments like electronic noses and hyperspectral imaging have proven effective at identifying food spoilage (Cao et al., 2023), (Capelli et al., 2014), (Sberveglieri et al., 2014), (Jia et al., 2022). A natural next step is to link the output of these sensors with machine learning models trained to detect unappealing textures and odors. Such predictive models have the broader potential to revolutionize quality assurance pipelines, ultimately increasing overall food safety and quality.

Here, we have explored the current state of data, models, and applications, and we have some palpable recommendations going forward. First, there are areas where more data are needed, especially when it comes to texture. Similarly, there are cases such as sourness or saltiness, where predictors are not readily available. Concomitantly, we need to move from reductionist models that predict a property in isolation in more composite predictors that predict the flavor and texture of complex foods. This can be achieved by integration of multiple predictors and datasets, in a way that it is more than the sum of the parts. Additionally, there is a lot of synergy that can come from bringing together mechanistic and statistical models, where the domain-agnostic statistical models can be the fabric on top of which the mechanistic, highly specific models can be built. For example, a model of chemical reactions like the Maillard reaction, can be supplemented with a neural network that connects parameters such as temperature, ingredients, and pressure, to outputs like appearance, flavor, and texture. Similarly computational fluid dynamics and finite element methods can dovetail well with machine learning methods that either learn the functions that map input to outputs, or they fine-tune the parameters within the mechanistic models, by superimposing and learning from the experimental trials. An integration of these computational methods with the production systems in the factory, especially after accounting for scale effects, will be transformative in our ability to create novel flavors, textures, and products in general.

## CRediT authorship contribution statement

**Michael Gunning:** Data curation, Formal analysis, Investigation, Visualization, Writing – original draft, Writing – review & editing. **Ilias Tagkopoulos:** Conceptualization, Funding acquisition, Project administration, Supervision, Writing – review & editing.

## Funding

The authors declare that financial support was received for the research, authorship, and/or publication of this article. This work was supported by the United States Department of Agriculture-National Institute of Food and Agriculture AI Institute for Next Generation Food Systems (AIFS), USDA-NIFA award number 2020–67021-32855.

## Declaration of competing interest

The authors declare that they have no known competing financial interests or personal relationships that could have appeared to influence the work reported in this paper.

## Data Availability

No data was used for the research described in the article.
